# Physical Properties of Slide-Ring Material Reinforced Ethylene Propylene Diene Rubber Composites

**DOI:** 10.3390/polym14102121

**Published:** 2022-05-23

**Authors:** Gyuri Kim, Pranabesh Sahu, Jeong Seok Oh

**Affiliations:** Department of Materials Engineering and Convergence Technology, RIGET, Gyeongsang National University, 501 Jinju-daero, Jinju 52828, Korea; rbfl1999@gnu.ac.kr (G.K.); pranab91@gnu.ac.kr (P.S.)

**Keywords:** slide-ring material, rubber composites, mechanical properties, compression set

## Abstract

High-damping rubber composites were prepared by mixing ethylene propylene diene monomer rubber (EPDM) with slide-ring (SR) materials using a two-roll mill, followed by a compression molding technique. SR material has a novel supramolecular structure with unique softness and slidable crosslink junctions. The mechanical strength, thermal stability, compression set property, and damping performance of the composites were investigated. The use of the high damping SR phase dispersed in the EPDM matrix displayed improved physical properties and damping performance compared to those of virgin rubber. As SR content increases in the composites, the damping factor of SR/EPDM blends becomes higher at room temperature. In addition to this, the SR composites showed excellent improvements in the compression set properties. The composites showed a compression set improvement of 35–38% compared to virgin EPDM. These improvements are due to the “pulley effect” of slide-ring materials. Therefore, these materials present a robust platform for making novel elastomer composites for high-performance damping and sealing applications.

## 1. Introduction

**Figure 1:**
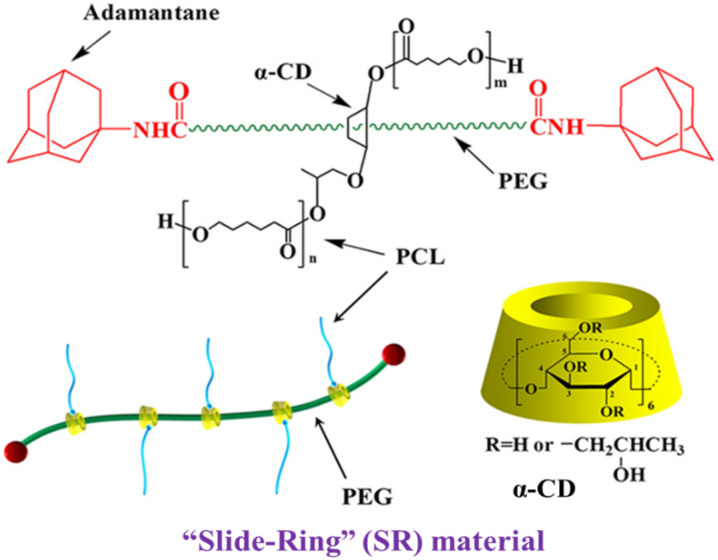
Structure of the slide-ring material. Reprinted from [13] with permission from Elsevier.

**Figure 2:**
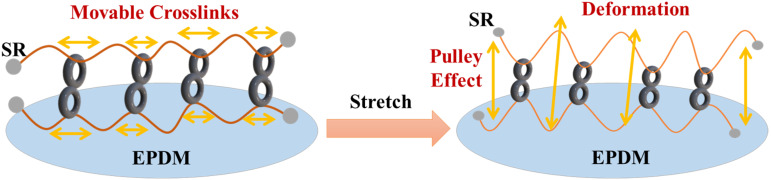
Schematics of ”Pulley effect” in slide-ring material-based composites before and after stretching.

“Slide-ring material (SR)”-based polymer composites are drawing considerable attention in the automobile industry and isolation bearings due to their high damping applications [1,2,3,4]. The SR material contains poly (ethylene glycol) in the main chains, with α-cyclodextrins (α-CD) grafted onto it. The α-CDs are modified by linear poly-ε-caprolactone chains, and the polymer backbones are blocked with bulky adamantine groups at both ends to prevent the detachment of α-CD rings [4,5,6]. In the SR structure, the polymer main chain is not fixed because the crosslinking points move freely, and the applied stress is evenly distributed throughout the material. This is called the ‘pulley effect’. Due to the pulley effect, polymer composites can exhibit a low compression set and significant loss factor [1,7,8]. The unique supramolecular structure of SR materials makes them useful as the preferred matrix for the preparation of high damping and stretchable rubber composites [9,10,11]. The chemical structure of SR and the mechanism of the “pulley effect” are shown in Figure 1 and Figure 2 respectively.

Research has been conducted on the preparation of rubber composites using SR material with significant mechanical strength and damping performance [12,13,14,15]. Wang et al. prepared natural rubber/SR material high damping composites for the first time [16]. Epoxidized natural rubber was used as a compatibilizer to disperse the high damping SR phase in the natural rubber matrix. Later, Wang et al. incorporated a sliding graft copolymer (SGC) into nitrile rubber to fabricate high-strength damping elastomers. The blends showed a distinct sea–island phase structure, good blend compatibility, and higher loss factors (tan δ) with increasing SGC content [17,18]. Highly dispersed graphene oxide (GO) was also used to fabricate an SR/GO composite as a stretchable base substrate for electronic applications [19].

The rubber material used in vibration isolators requires high damping abilities and flexibility to absorb the applied stress and resist any damage [20,21,22,23,24,25]. Unlike conventional rubbers, SR is a new material with cross-linking junctions on α-CDs rings that can freely slide along the backbone. The slidability of these cross-linking points leads to a softer mechanical response compared to material with fixed cross-links. Ethylene-Propylene Diene Monomer Rubber (EPDM) is one of the most widely used synthetic rubbers for automobile and other outdoor applications due to its high rebound elasticity, high strength, low compression set, and good low-temperature characteristics [26,27]. However, EPDM rubber exhibits low damping properties because the energy dissipation capability is poor [28]. Therefore, to improve the damping property for isolation bearings or automotive applications, an effective way of developing rubber composites using chemical modification and physical blending is highly desired. The application of SR material to EPDM rubber for damping applications has not been reported to date.

In this study, an elastomer composite was developed using a slide-ring material and dispersed in the EPDM rubber matrix following a simple internal-mixing process for high-damping applications. A slide-ring material was introduced into EPDM as the high damping phase to prepare all the rubber composites. The morphological and mechanical properties of the composites were investigated using an atomic force microscope (AFM), and tensile testing. For a good damping performance, a dynamic mechanical (DMA) response i.e., loss factor (tan δ) of the composites, was determined with temperature and frequency. The effect of SR on the compression set property of SR/EPDM composite, which is an important part of this investigation, was determined. These results indicate that the composites can be potentially used as deformable base substrates for isolation bearings and automotive parts.

## 2. Material and Methods

### 2.1. Materials

EPDM rubber (KEP330) was purchased from KUMHO Polychem., Daejeon, Korea. Slide-ring material (SH2400P) was procured from Advanced Soft Materials Inc. (Tokyo, Japan). Reagent grade -zinc oxide (>99.0% assay) and stearic acid were obtained from Daejung Chem., Seoul, Korea. Sulfur (99.5%) of 325 mesh size was supplied by Thermo-Fisher Scientific, Daejeon, Korea. Other compounding ingredients, such as 2-mercaptobenzothiazole (MBT) and tetramethyl thiuram disulfide (TMTD), were purchased from Sigma-Aldrich, Seoul, Korea and used as received.

### 2.2. Preparation of EPDM/Slide-Ring Composites

**Figure 3:**
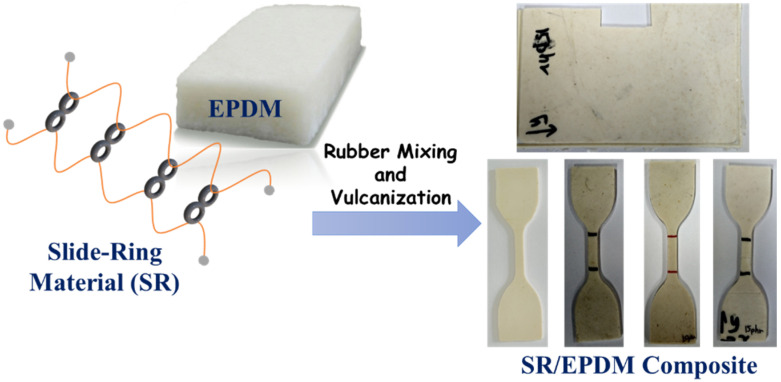
Schematic illustration of rubber composites’ formation using dynamic vulcanization.

The compounding formulation for the preparation of SR/EPDM composites is given in Table 1. At first, the slide-ring material was dried in an oven for 2 h at 175 °C to prepare the pre-crosslinked poly-ε-caprolactone (PCL) chains. EPDM rubber was passed into a two-roll mill first for 1–2 min before SR was mixed. Thereafter, compounding ingredients were added and mixed onto the two-roll mill at a front roll speed of 45 rpm. At last, sulfur was added to the SR/EPDM composites. The total mixing time was 10 min. Finally, the compounds were vulcanized by compression molding at 160 °C and 15 MPa for 10 min optimum cure time, as obtained from the rheometer. The schematics for the formation of rubber composites are shown in Figure 3.

### 2.3. Characterization Methods

#### 2.3.1. Compression Set

The compression set test was performed according to ASTM D395 [29] on a standard test specimen of cylindrical shape (29 ± 0.5 mm diameter and 12.5 ± 0.5 mm thickness) vulcanized by the compression molding method. The test specimen was placed between the plates of the compression device with the spacers on each side. The percentage of the compression employed was 25% of the original thickness. Then, the assembled compression device was placed in an oven at 70 °C for 70 h and allowed to cool for 30 min for a final thickness measurement. The compression set is measured as follows:

(1) Compression set (%)=t0−t1t0−ts×100

where $t_{0}$ = the original thickness of the sample; $t_{1}$ = the thickness of the sample after removal from the device; and $t_{s}$ = thickness of the spacer bar used.

#### 2.3.2. Dynamic Mechanical Analysis (DMA)

The dynamic mechanical properties, i.e., damping performance were performed using a dynamic mechanical analyzer (DMA Q800, TA instrument, New Castle, DE, USA). The specimens were cut to be 20 mm long, 5 mm width, and 2 mm thick. The temperature dependence of the damping or loss factor (tan δ) was measured between −70 and 100 °C at a heating rate of 3 °C/min, 10 Hz frequency, and 10 μm amplitude. A dynamic frequency sweep was carried out from 0.1 to 15 Hz at 10 μm amplitude at 30 °C. For isolation bearings’ application, tan δ values greater than 0.1 in the 0.2–5 Hz frequency range are highly desired, according to the ISO standard [30].

#### 2.3.3. Mechanical Properties

The mechanical properties were measured following ISO 37 by the universal testing machine (Tensometer 2000, Myungjitech, Yongsan-gu, Seoul, Korea) at a crosshead speed of 500 mm/min. The dumbbell-shaped samples (25 mm × 5 mm × 2 mm) were prepared according to ISO 37 Type 1A. An average value of five specimen measurements was reported.

#### 2.3.4. Thermogravimetric Analysis (TGA)

The thermal analysis was carried out using Q600 TA instruments (New Castle, DE, USA) and the test samples were cut from the vulcanized sample (5–10 mg). The specimen was heated from 50 °C to 800 °C at a constant temperature (10 °C/min) in a nitrogen atmosphere and the weight loss was determined as a function of temperature.

## 3. Results and Discussion

### 3.1. Compression Set

**Figure 4:**
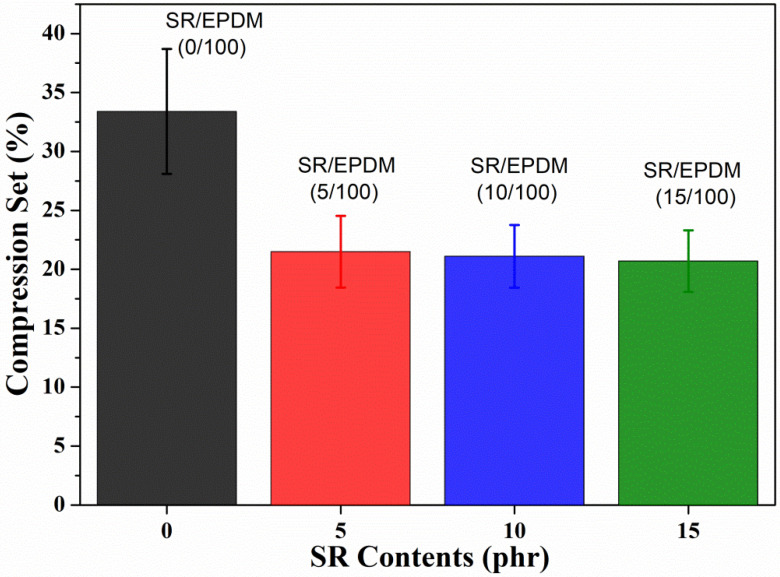
Compression set of SR/EPDM composites with varying SR contents.

The compression set is an important property for rubber sealing applications, as spontaneous stress variation occurs due to internal pressure or external forces. Figure 4 shows the bar graph of the compression set property of SR/EPDM composites. The percentages of improvements in the compression set with increasing SR content (compared to virgin EPDM) are given in Table 2. The compression set of virgin EPDM was obtained at about 33.4%. With the incorporation of SR material in the composites, there is a significant reduction in compression set values with respect to virgin EPDM, i.e., good compression set behavior. At higher loadings of 15 phr, SR shows good compatibility with the elastomer matrix, which leads to a decrease in the compression set value of the SR/EPDM (15/100) composites. The change in C-set values is not as significant for different SR contents (5 phr, 10 phr, and 15 phr) composites. However, compared with virgin EPDM, it can be seen that, when SR is added, the compression set is significantly reduced. The lower the percentage, the better the material resists permanent deformation under certain external forces [31,32]. The percentage of C-Set property improvement for SR-based composites is observed to be around 35–38% when compared with the virgin EPDM value. This means that the deformation rate is lower for SR/EPDM composites and adequate for sealant or high damping applications.

### 3.2. Dynamic Mechanical Analysis

**Figure 5:**
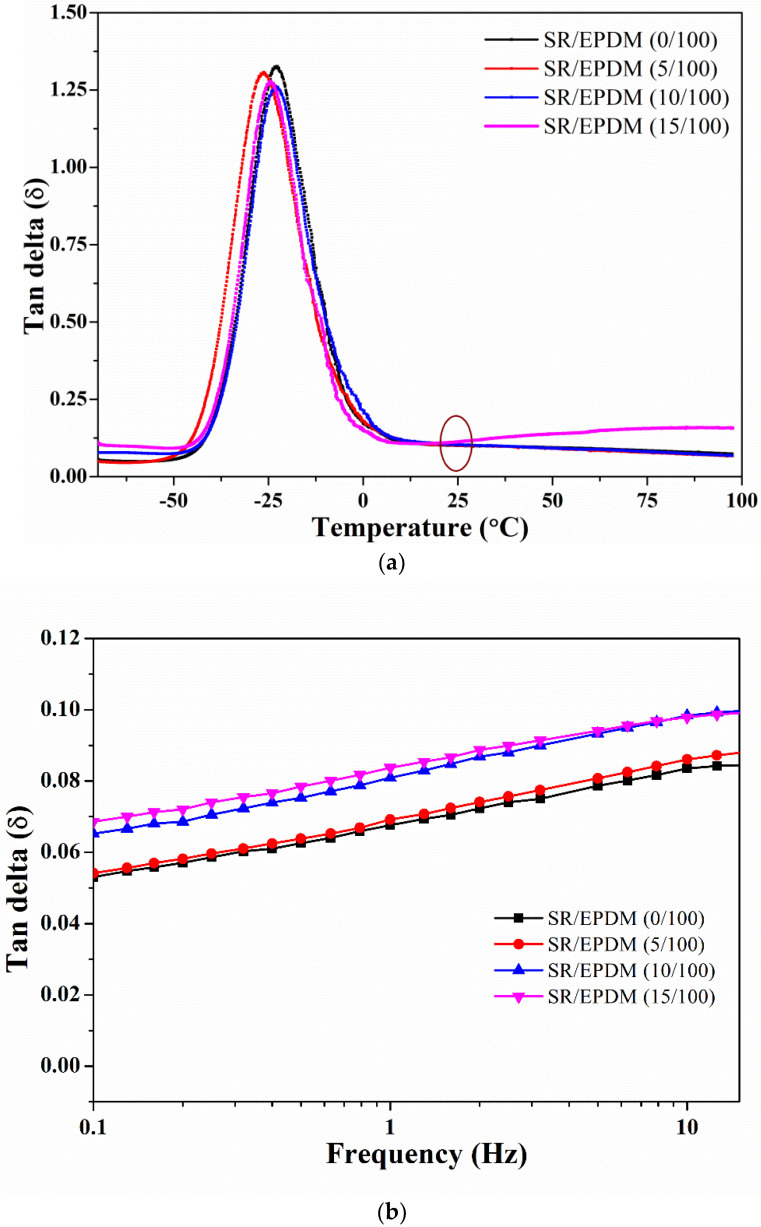
(**a**) Temperature dependence of tan δ and (**b**) variation of tan δ with dynamic frequency sweep for SR/EPDM composites.

Figure 5a shows the temperature dependence of tan δ for the SR/EPDM composites. For good damping performance, a high and broad tan δ peak is required [16]. A broad tan δ peak is observed around -25 °C is observed for all the SR/EPDM composites. The value of tan δ is almost the same for virgin EPDM, 5 phr, and 10 phr SR/EPDM composites at room temperature. However, in the case of 15 phr SR/EPDM composites, the tan δ value is highest at room temperature. This phenomenon can be attributed to the presence of the damping phase (SR), which shows the “pulley effect”, resulting in a higher tan δ value [16,18]. The high tan δ value of SR/EPDM (15/100) blends signify a good damping performance. The tan δ values of SR/EPDM (0/100) and SR/EPDM (15/100) are 0.10 and 0.11 at room temperature, respectively.

The damping performance of SR/EPDM composites under dynamic frequency sweep was also conducted, as shown in Figure 5b. Dynamic frequency sweep was carried out from 0.1 Hz to 15 Hz at a constant amplitude of 10 μm at 30 °C. The SR/EPDM (5/100) composite possesses a higher tan δ value than virgin EPDM. As the SR content increases, the tan δ values distinctly increase with increasing frequency. These results indicate a better damping performance from SR/EPDM composites at higher frequencies. Overall, the improved damping properties at high temperatures and frequencies have potential applications in automobile anti-vibration parts.

### 3.3. Mechanical Properties

**Figure 6:**
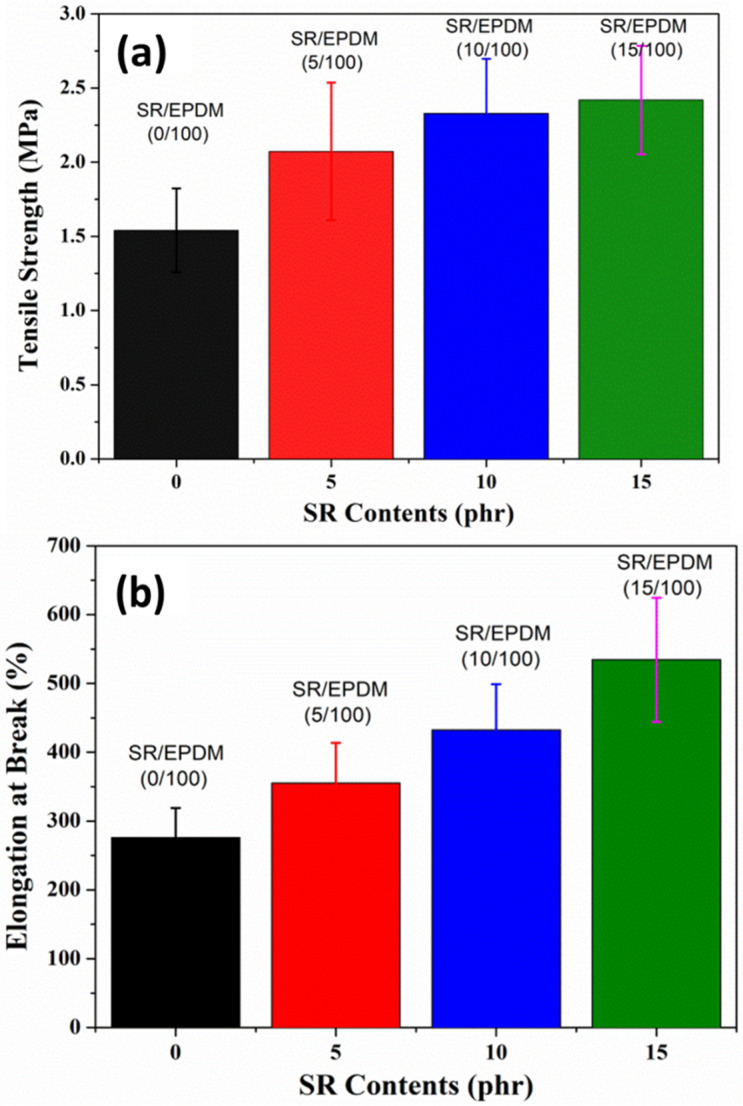
(**a**) Tensile strength; and (**b**) Elongation at break of SR/EPDM composites with different SR contents.

The effect of SR on the tensile strength and elongation at break of SR/EPDM composites are shown in Figure 6. The tensile strength of virgin EPDM is 1.54 MPa. However, the composites with SR loading showed an increase in mechanical properties, i.e., tensile strength, compared to neat EPDM. The tensile strength of SR/EPDM composites for 5 phr, 10 phr, and 15 phr were obtained at 2.07 MPa, 2.33 MPa, and 2.42 MPa, respectively. The SR/EPDM composite (15/100) exhibited the highest tensile strength of 2.42 MPa. The percentage of tensile strength improvement for SR-based composites is observed around 34–57% compared to virgin EPDM. With the increase in SR loading, the elongation at break of SR/EPDM composites is also significantly improved. The SR/EPDM composite (15/100) showed the highest elongation at break at 534%. This suggests that both tensile strength and elongation increased with increasing SR contents, indicating the outcome of the pulley effect in the composites.

### 3.4. Thermal Analysis

**Figure 7:**
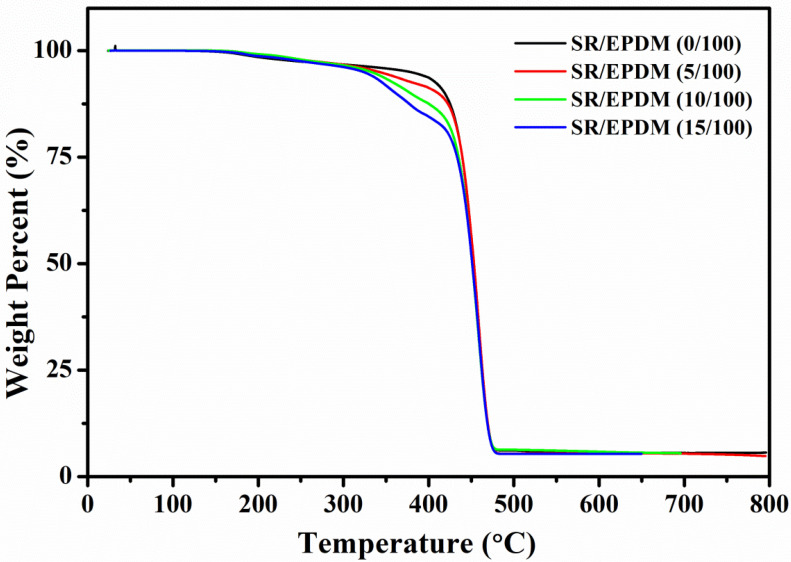
Thermogravimetric analysis of the SR/EPDM composites.

The thermal stability of the SR/EPDM elastomer composites as a function of temperature is measured using thermogravimetric analysis (TGA), as represented in Figure 7. All the SR/EPDM blends displayed two distinct stages of thermal degradation, from 160 °C to 320 °C and 320 °C to 420 °C, respectively. The drop-in rate of weight loss increases with increasing SR content, reaching a maximum for SR/EPDM (15/100) bends.

## 4. Conclusions

In this study, slide-ring material/EPDM rubber composites were fabricated using simple rubber mixing and compounding procedure. The addition of a high damping SR phase in the rubber matrix showed a significantly improved damping performance and good mechanical strength. The tensile strength and elongation at break of the SR/EPDM composites improved compared to virgin rubber, due to the sliding nature and pulley effect of the slide-ring material. Most importantly, with the addition of SR, the composites showed improved compression set properties. From the dynamic mechanical results, the room temperature loss factor, i.e., tan δ increased with an increase in SR content at a dynamic frequency and temperature conditions for the composites. The improved damping property of the composites makes it suitable for application in automobile parts where vibration protection is essential, such as engine mounts and bushes. On the other hand, as the compression set decreases, it is expected to be used for oil seals, weather strips, etc.

## Figures and Tables

**Table 1:** Typical compounding formulation of SR/EPDM composites.

Ingredients	Contents (phr) ^a^
EPDM	100
SR	0, 5, 10, and 15
Zinc Oxide	5.0
Stearic acid	1.0
2-Mercaptobenzothiazole (MBT)	0.5
Tetramethyl thiuram disulfide (TMTD)	1.0
Sulfur	1.5

^a^ phr: parts per hundred parts of rubber by weight.

**Table 2:** Compression set values of SR/EPDM composites.

SR Contents	% of C-Set Improvement
0 phr	-----
5 phr	35.6
10 phr	36.8
15 phr	38.0

phr: parts per hundred parts of rubber.

## Data Availability

The data presented in this study are available upon request from the corresponding author.
